# Rehabilitation in progressive supranuclear palsy: Effectiveness of two multidisciplinary treatments

**DOI:** 10.1371/journal.pone.0170927

**Published:** 2017-02-03

**Authors:** Ilaria Clerici, Davide Ferrazzoli, Roberto Maestri, Fabiola Bossio, Ilaria Zivi, Margherita Canesi, Gianni Pezzoli, Giuseppe Frazzitta

**Affiliations:** 1 Department of Parkinson’s disease, Movement Disorders and Brain Injury Rehabilitation, “Moriggia-Pelascini” Hospital, Gravedona ed Uniti, Italy; 2 Department of Biomedical Engineering, Istituti Clinici Scientifici Maugeri, Scientific Institute of Montescano, Montescano, Italy; 3 Parkinson Institute, Istituti Clinici di Perfezionamento, Milano, Italy; University of Toronto, CANADA

## Abstract

**Background:**

to date, there are no medical or surgical treatments for progressive supranuclear palsy (PSP). It is possible to speculate that patients with PSP could benefit from rehabilitative treatments designed for Parkinson’s disease, including the use of robot-assisted walking training.

**Objective:**

to evaluate whether the use of the robotic device Lokomat® is superior in PSP patients to the use of treadmill with visual cues and auditory feedbacks (treadmill-plus) in the context of an aerobic, multidisciplinary, intensive, motor-cognitive and goal-based rehabilitation treatment (MIRT) conceived for Parkinsonian patients.

**Methods:**

we enrolled twenty-four PSP patients. Twelve subjects underwent a 4-week MIRT exploiting the use of the treadmill-plus (MIRT group). Twelve subjects underwent the same treatment, but replacing the treadmill-plus with Lokomat® (MIRT-Lokomat group). Subjects were evaluated with clinical and functional scales at admission and discharge. The primary outcomes were the total PSP Rating Scale (PSPRS) score and its “limb” and “gait” sub-scores. Secondary outcomes were Berg Balance Scale (BBS), Six Minutes Walking test (6MWT) and the number of falls.

**Results:**

total PSPRS, PSPRS-gait sub-score, BBS, 6MWT and number of falls improved significantly in both groups (p ≤ 0.003 all, except 6MWT, p = 0.032 and p = 0.018 in MIRT-Lokomat and MIRT group respectively). The PSPRS-limb sub-score improved significantly only in the MIRT group (p = 0.002). A significant difference between groups was observed only for total PSPRS, indicating a slightly better improvement for patients in the MIRT group (p = 0.047). No differences between groups were revealed for the other outcomes, indicating that the effect of rehabilitation was similar in both groups.

**Conclusions:**

Lokomat**®** training, in comparison with treadmill-plus training, does not provide further benefits in PSP patients undergoing MIRT. Our findings suggest the usefulness of an aerobic, multidisciplinary, intensive, motor-cognitive and goal-based approach for the rehabilitation of patients suffering from such a complex disease as PSP.

**Trial Registration:**

This trial was registered on ClinicalTrials.gov, NCT02109393.

## Introduction

Progressive supranuclear palsy (PSP) represents the most common form of atypical Parkinsonism[1], with a prevalence of 6.5 cases/1.000.000 people.[2] The neuropathological hallmark of PSP is a biochemical alteration in the tau protein, which results in a neurodegeneration and gliosis in the basal ganglia, brainstem, prefrontal cortex and cerebellum.[3]

Clinical features of PSP include early postural instability with recurrent falls (mostly backwards), speech problems, swallowing difficulties, visual dysfunctions (vertical supranuclear gaze palsy), pseudobulbar palsy, bradykinesia, axial rigidity and neuropsychological deficits.[4]Falls represent the main cause of reduced independence, morbidity and mortality in this disorder.[5]

There are no effective medical or surgical treatments for PSP. Rehabilitative interventions are described in sporadic case reports[6–8] or in few studies with limited sample sizes[9,10] and consist of exercise programs focused to improve muscle strength, gait, coordination and balance.[6–11] Nevertheless, data from these studies are not consistent and no specific rehabilitation treatment exists for this disease.[4]

Conversely, several authors support the efficacy of aerobic, intensive and/or multidisciplinary rehabilitation treatments on gait and balance in patients affected by Parkinson’s Disease (PD).[12–22]

Although PSP and PD represent independent processes, some neuropathological data provide evidences about a common deficit in both pathologies.[23,24] Therefore, we could speculate that some treatment modalities successfully used to improve motor performances in PD patients could also be useful in people with PSP.

Although already shown that an intensive exercise program is feasible, safe and could help people with PSP[8], the severe gait and balance disturbances, together with the high risk of falls, limit the patients’ participation to the conventional physiotherapy. Some authors partially overcame this difficulty by integrating the physical treatment with the use of supported treadmills[6] or robot-assisted walking trainings.[11] Furthermore, some data suggest that they help to improve the gait and reduce the risk of falls in other neurological disorders.[11,25] Nevertheless, the high cost of robotic devices raises the question of their cost/benefit ratio.

Several studies have demonstrated the effectiveness of a treadmill training in improving gait parameters (such as gait speed and stride length) and balance in subjects with PD.[26,27] On the other hand, little evidence has been found on the effects of this device on tauopathies[6–8], including PSP.

The aim of our study was to evaluate whether, in patients affected by PSP, a robotic device (Lokomat®) is superior to a treadmill with visual cues and auditory feedbacks (treadmill-plus) in the context of an aerobic, multidisciplinary, intensive, motor-cognitive and goal-based rehabilitation treatment conceived for PD.

## Methods and materials

### Subjects

We enrolled twenty-four consecutive PSP patients admitted from April 2014 to December 2015 to the Department of Parkinson’s Disease, Movement Disorders and Brain Injury Rehabilitation of the “Moriggia-Pelascini” Hospital (Gravedona ed Uniti, Italy) for a 4-week Multidisciplinary Intensive Rehabilitation Treatment (MIRT). Inclusion criteria were: a) diagnosis of PSP in accordance to the NINDS-SPSP International Criteria[5], b) ability to walk without assistance for at least 6 meters, c) stable dopaminergic drugs dosage in the month preceding the admission. Exclusion criteria were: a) any other significant neurological or orthopedic disorder, b) osteoarthritis, osteoporosis, cutaneous lesions and/or other pressure wounds, c) body weight ≥135 kg (upper limit for the use of Lokomat®), d) respiratory and cardiovascular diseases.

All participants reported falls in the month preceding the admission to the hospital.

Patients were randomly assigned to one of two groups (each one composed of 12 patients) using a computer generated list: i) group 1 (MIRT group) underwent a 4-week MIRT exploiting the use of a treadmill associated with visual cues and auditory feedbacks (treadmill-plus), as previously described in PD patients[16]; ii) group 2 (MIRT-Lokomat group) underwent a 4-week MIRT replacing the treadmill-plus with Lokomat®.

The study was approved by the local Scientific Committee and Institutional Review Board (‘Moriggia-Pelascini’ Hospital) and was in accordance with the code of Ethics of the World Medical Association (Declaration of Helsinki, 1967). A complete explanation of the study protocol was provided and a written informed consent was obtained from all patients before they began their participation in this study. This trial was registered on ClinicalTrials.gov, NCT02109393.

### Rehabilitation treatment

MIRT has been described in previous papers.[14,15] It is an aerobic, multidisciplinary, intensive, motor-cognitive and goal based rehabilitation treatment. It consists of a 4-week physical therapy in a hospital setting and entails four daily sessions for five days per week. Different healthcare professionals are involved in this protocol: Neurologists, Physiatrist, Physiotherapists, Occupational therapists, Speech therapists, Nurses, Neuropsychologist and Nutritionist. All treatments are performed in an aerobic condition and with an exercise intensity of 70–80% of the heart rate reserve. The duration of each session, including recovery periods, is about one hour. The first daily session consists of a one-to-one session with a physical therapist: it comprises cardiovascular warm-up activities, relaxation, muscle-stretching exercises to improve the range of motion of spinal, pelvic and scapular joints, activities to improve the functionality of the antigravity muscles and exercises to improve motor abilities during postural changes. The second daily session includes aerobic and repetitive activities to improve gait and balance using different devices: specifically, treadmill-plus[16] or Lokomat®, cycloergometer, crossover[17] and posturographic platform with visual feedbacks. The third is a daily session of occupational therapy to improve autonomy in activities of everyday life. The last daily session includes one hour of speech therapy.

#### Mirt group

During the second session, all patients in the MIRT group underwent 20-minutes treadmill-plus training per day (Gait Trainer 3 Biodex, Biodex Medical System– 20 Ramsay Road, Shirley, New York, USA), 5 times a week, for 4 weeks. A physiotherapist expert in movement disorders supervised the patients during the treadmill training. Maximum tolerated walking speed was determined for each patient at the beginning of the first session. Treadmill speed was initially set at 1.0–1.5 km/h and progressively increased until a maximum of 2.5 km/h, depending on the patients’ physical abilities. During the training, visual cues and auditory feedbacks were used. The visual cue was a target, defined by two horizontal lines displayed on a screen that the patient had to reach with the stride. The space between the two lines was personalized for each patient according to gender, height and age. The right and left footprints were shown alternatively on the screen: when they fell within the two lines, a ‘‘well done” message appeared on the screen; otherwise, patients were invited to take a longer or shorter step in order to adapt the stride length to the set target. The auditory feedback consisted of musical beats synchronized with the visual cues.

#### Mirt-lokomat group

During the second session, patients in the MIRT-Lokomat group underwent 20-minutes Lokomat® training per day, 5 times a week, for 4 weeks. Lokomat® (Hocoma AG, Industriestrasse 4, CH 8604 Voketswil, Switzerland) is a driven-gait orthosis (gait robot) that allows gait on a treadmill by simulating the human physiological stride pattern. As for the treadmill-plus training, the belt speed was set at the maximum velocity tolerated by each patient, not exceeding 2.5 km/h. The driven orthosis is endowed with electrical drives in knee and hip joints with four force transducers with 4 amplifiers. The orthosis is adaptable to subjects according to femur lengths (trochanter to knee joint cavity, from 35 to 47 cm) and pelvic width (from 29 to 51 cm).

### Outcome measures

All patients were evaluated at admission (T0) and discharge (T1), at 9 AM, by neurologists and physiotherapists with expertise in movement disorders. Outcomes assessors were blind to group allocation and study design.

#### Primary outcome

The primary outcome was the PSP rating scale (PSPRS), of which we analyzed the total score and the scores obtained in the “limb” and “gait” sub-sections.[28]

#### Secondary outcome

The secondary outcomes were Berg Balance Scale (BBS), Six Minutes Walking test (6MWT),[29] and number of falls. BBS[30] consists of 14 items designed to rate balance in sitting, standing, turning, and reaching forward. Each item is rated from 0 to 4, with a maximum score of 56 (best score). A rating of 0 means either assistance needed or unable to perform task. The test-retest reliability (ICC = 0.98) for BBS was reported in patients with parkinsonism.[31] The 6MWT assesses the distance walked over 6 minutes, as a sub-maximal test of aerobic capacity/endurance. 6MWT has been shown to have excellent test-retest reliability (ICC = 0.95–0.96) for subjects with PD.[31] There are no reliability values for this test in PSP patients.

A fall was defined as an unexpected event that results in the patient inadvertently resting on the floor.[32] It had not to be the result of a blow, loss of consciousness, sudden onset of paralysis, or epileptic seizure.[32] Patients were asked to complete a questionnaire concerning the falls incidence in the week preceding the admission, and during the last week of the rehabilitation treatment. The questionnaire consisted of 2 open-ended questions related to the number and time of falls.

### Statistical analysis

#### Sample size computation

Given the difficulties in finding patients fulfilling the study inclusion criteria, we chose a sample size of convenience (12+12 patients) based on the average number of potentially eligible patients admitted to our Hospital in a two-year period. As a matter of fact, sample size computation based on the primary outcome measure, namely PSPRS, was not feasible since we are not aware of measures of standard error of measurement (SEM) reported in the literature for this variable. Carrying out a power calculation based on the secondary outcome measure BBS for the chosen sample size with SEM equal to 1.8,[31] minimum difference between the effect of treatments that we wanted to detect equal to 3, two-tailed type I error of 0.05, the computed power was 0.83 (normal approximation, two sample t-test).

#### Data analysis

The normality of the distribution of all variables was assessed by the Shapiro–Wilk test.

Descriptive statistics for continuous variables are reported as median (lower quartile, upper quartile) or mean±SD for non-normally and normally data respectively and as number (frequency percentage) for discrete variables. For non-normally distributed variables, between- and within-group comparisons were performed by the Mann-Whitney U test and Wilcoxon signed-rank test respectively. The unpaired and paired t-tests were used for normally distributed variables. Comparisons of categorical variables were carried out by the Chi-square test or Fisher exact test when appropriate.

Most outcome variables were non-normally distributed. To assess whether the MIRT-Lokomat protocol could lead to a better improvement as compared to MIRT, for each outcome variable we computed the difference (discharge-admission) and then ran the non parametric test on the treatment factor.

The standardized mean difference (Cohen’s d) was also computed and used to assess the effect size.

All statistical tests were two-tailed and statistical significance was set at p < 0.05.

All analyses were carried out using the SAS/STAT statistical package, release 9.2 (SAS Institute Inc., Cary, NC, U.S.A.).

## Results

**Fig 1:**
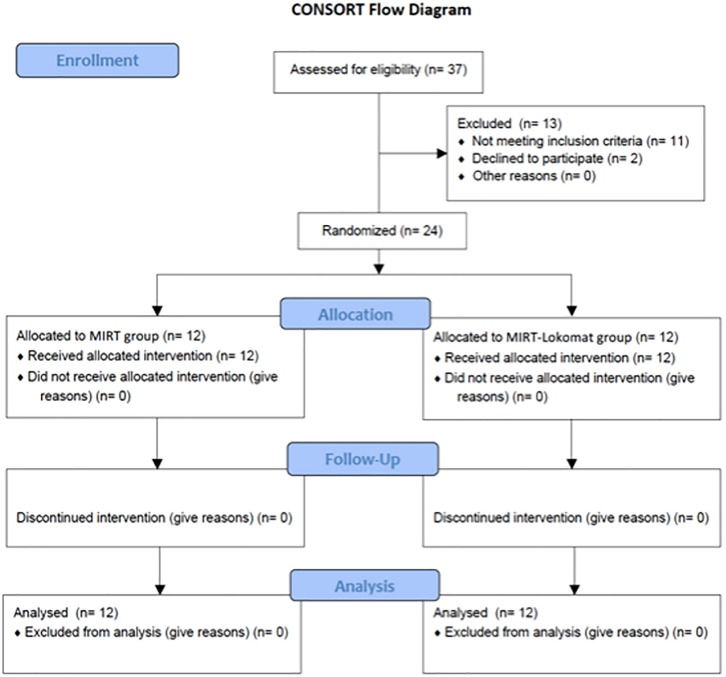
CONSORT flow diagram.

Fig 1 (CONSORT flow diagram) shows the trial profile. No dropouts were recorded during the treatment and all subjects completed the rehabilitative protocols.

The demographic and clinical characteristics of patients assigned to MIRT group and MIRT-Lokomat group are reported in Table 1. No statistically significant differences were observed at baseline between the two groups in any of the different variables.

**Table 1:** Demographic and clinical characteristics of patients assigned to MIRT group and MIRT-Lokomat group. Reported p-values are computed by the Chi-square test for the variable Sex, by unpaired t-test for age, LED, weight, height, disease duration and by the Mann–Whitney U test for all the other variables. Data are reported as median (lower quartile, upper quartile) or mean±SD for non-normally and normally data respectively and as number (frequency percentage) for discrete variables.

Variable	MIRT-Lokomat group	MIRT group	p-value
**Age (years) Range**	69.9±5.2 60–77	72.5±6.1 64–83	0.28
**LED (mg/die)**	274.2 ± 217.9	375.8 ± 254.5	0.31
**Sex (% Male)**	41	58	0.41
**Weight (Kg)**	71.4±5.3	71.9±6.4	0.84
**Height (cm)**	169±7.1	168±5.9	0.84
**Disease duration (yrs)**	4.1 ± 1.4	4.0 ± 1.2	0.88
**MMSE**	25.6±1.94	25.1±3.87	0.69
**FAB**	11.7±3.7	10.5±2.48	0.46
**PSPRS-Total**	35.0 (29.0,44.5)	34.0 (25.0,42.0)	0.47
**PSPRS-limb**	5.50 (3.50,6.00)	5.00 (3.50,6.50)	0.98
**PSPRS-gait**	12.0 (9.0,13.0)	10.5 (8.5,13.5)	0.91

Abbreviations: MIRT (Multidisciplinary Intensive Rehabilitation Treatment); LED (levodopa equivalent dose), FAB (Frontal Assessment Battery); MMSE (Mini Mental State Examination); PSPRS (Progressive Supranuclear Palsy Rating Scale).

Baseline (T0) and end of treatment (T1) values of outcome variables are reported in Table 2 for both groups. Regarding the number of falls, T0 and T1 correspond to falls incidence in the week preceding the respective observation time. Total PSPRS, PSPRS-gait, BBS, 6MWT and number of falls improved significantly by the end of the training programs in both groups. The corresponding effect sizes (standardized differences) were: -0.6, -1.5, 1.2, 0.5, -2 for MIRT-Lokomat group and -0.6, -1.3, 1.4, 0.4, -1.3 for MIRT group. Conversely, PSPRS-limb improved significantly only in the MIRT group (p = 0.0020, effect size = -0.7).

**Table 2:** Baseline and end of treatment values of outcome variables for both groups of patients. Reported p-values are computed by the Wilcoxon signed rank test. Data are reported as median (lower quartile, upper quartile).

Variable	MIRT-Lokomat group T0	MIRT-Lokomat groupt T1	p-value	MIRT group T0	MIRT group T1	p-value
**PSPRS-Total**	35.0 (29.0,44.5)	31.5 (25.5,38.0)	0.0005	34.0 (25.0,42.0)	27.5 (19.0,32.0)	0.0005
**PSPRS-limb**	5.50 (3.50,6.00)	4.00 (3.00,5.00)	0.076	5.00 (3.50,6.50)	4.00 (2.00,4.00)	0.0020
**PSPRS-gait**	12.0 (9.0,13.0)	8.5 (7.0,10.0)	0.0005	10.5 (8.5,13.5)	7.5 (5.0,9.5)	0.0005
**BBS**	30.0 (26.5,38.5)	47.0 (35.5,51.5)	0.0005	35.5 (25.0,43.5)	49.0 (45.5,51.0)	0.0005
**6MWT**	223 (189,282)	270 (232,325)	0.032	262 (176,322)	276 (236,349)	0.018
**Number of Falls**	8.00 (5.00,10.50)	2.00 (1.00,2.00)	0.0015	7.50 (5.50,10.50)	2.00 (1.00,2.50)	0.0029

Abbreviations: MIRT (Multidisciplinary Intensive Rehabilitation Treatment); PSPRS (Progressive Supranuclear Palsy Rating Scale); BBS (Berg Balance Scale); 6MWT (Six Minutes Walking test).

Finally, Table 3 reports the difference (discharge-admission) of the outcome variables for MIRT-Lokomat group and MIRT group. A significant difference was observed only for total PSPRS (p = 0.047), indicating a slightly better improvement for patients in the MIRT group. No difference was revealed for the other outcome variables, indicating that the effect of rehabilitation was similar in both groups.

**Table 3:** Difference (discharge-admission) of the outcome variables for MIRT+Lokomat group and MIRT group and effect size. Reported p-values are computed by the Mann–Whitney U test.

Variable	delta–MIRT-Lokomat group	Effect size	delta–MIRT group	Effect size	p-value
**PSPRS-total**	-5.00 (-5.50,-3.50)	-0.6	-8.00 (-9.50,-5.00)	-0.6	0.047
**PSPRS-limb**	-1.00 (-1.00,-0.50)	-0.4	-1.50 (-2.00,-1.00)	-0.7	0.067
**PSPRS-gait**	-3.00 (-4.00,-2.00)	-1.5	-4.00 (-4.50,-3.00)	-1.3	0.17
**BBS**	9.50 (6.50,19.00)	1.2	10.50 (8.00,20.50)	1.4	0.40
**6MWT**	32.5 (19.5,99.5)	0.5	55.5 (-3.0,67.5)	0.4	0.62
**Number of Falls**	-6.50 (-8.00,-3.50)	-2	-5.50 (-9.00,-3.50)	-1.3	0.98

Abbreviations: MIRT (Multidisciplinary Intensive Rehabilitation Treatment); PSPRS (Progressive Supranuclear Palsy Rating Scale); BBS (Berg Balance Scale); 6MWT (Six Minutes Walking test).

## Discussion

The main finding of this study is that a specific aerobic, intensive, motor-cognitive, goal-based and multidisciplinary rehabilitation protocol improves the total PSPRS, PSPRS-gait, BBS and 6MWT scores and the number of falls in PSP patients.

The improvements we found in PSPRS-gait and 6MWT indicate that both treadmill-plus and Lokomat®, used in the context of MIRT, provide benefits on gait in PSP patients.

The strength of these improvements was remarkable in the light of computed effect sizes (all from moderate to high) and of measures of minimally clinically important differences reported in the Rehabilitation Measures Database (www.rehabmeasures.org) in other populations of patients (34.4 m for 6MWT).[33] Our results confirm the findings from previous studies demonstrating the effectiveness of the treadmill training on gait and balance of patients affected by PD and PSP.[6,26,27,34] Egerton T et al[24] showed that subjects with PSP share the same defective scaling in stride length that underlines gait disturbances in PD: this fact is consistent with the presence of a mismatch with the stride length selection associated with basal ganglia malfunction in both diseases.[24] Therefore, we can assume that the use of treadmill-plus, which is successful in improving gait in people with PD, may be appropriate also for subjects with PSP.[24] Other factors can reasonably explain why patients in the MIRT-Lokomat group achieved functional improvements similar to those observed in subjects in the MIRT group: first, Lokomat® allows patients to walk repetitively, resembling the over-ground gait and exploiting proprioceptive and exteroceptive feedbacks.[35] Second, a robotic gait training can overcome all the limitations about the repeatability of the movement with respect to the human-human interaction.[11]

The decrease in the number of falls, together with the improvement of BBS score in our patients, confirms that both treatments improve the balance dysfunction. This is an interesting point, since falls is the main cause of reduced independence, morbidity and mortality in PSP[5] and the control of this phenomenon should become the main goal of any rehabilitative approach designed for this group of patients. We conceive that patients improved in those spatio-temporal gait parameters mostly connected with the risk of falls (cadence, step length, stride length, velocity and step width)[36] following both treatments. Although we have not evaluated these measures in our patients, some authors have already shown that a robot-assisted gait training[11] or a supported treadmill training[6] lead to positive effects on spatio-temporal gait parameters in subjects with PSP. Moreover, it must be considered that patients in both groups received a posturographic platform balance training. During the training sessions, while looking at the screen connected to the posturographic platform, patients have to follow pathways of different shapes and lengths by using a cursor sensitive to the displacement of the center of gravity given by their feet movements on the platform. Patients with atypical parkinsonism (including PSP) suffer from balance impairment principally in the medio-lateral plane.[37] For that reason, specific balance exercises are performed on the posturographic platform to reduce the body sway along the medio-lateral axis. Therefore, the use of the posturographic platform, together with treadmill-plus or Lokomat® training, could have contributed to the balance improvement and the decrease in rate of falls.

Finally, we found that the PSPRS-limb score improved significantly only in MIRT group. Treadmill-plus training exploits an active involvement of the trunk muscles. This is different to what happens during Lokomat ® training, since patient’s weight is supported by the exoskeleton. A good trunk control is linked with better limb functionality;[38–41] therefore, we argue that the use of treadmill-plus could improve limbs functioning, as it exploits an active trunk activity. On the other hand, this condition is not satisfied when the patient is trained with Lokomat® because the exoskeleton supports the patient’s weight and the trunk control is passively maintained.

This study confirms the importance of an aerobic, multidisciplinary, motor-cognitive, goal-based and intensive approach for the rehabilitation of patients suffering from such a complex disease as PSP. Our data are similar to those from other studies showing that patients with atypical parkinsonism benefit from an inpatient interdisciplinary movement disorders program to improve their functional status.[42] Furthermore, other previous small series described rehabilitation as a possible therapeutic approach for PSP.[6,8,11] Treatments like MIRT and MIRT-Lokomat, stimulating the selective attention processes through the use of cues, feedbacks and motivation, probably work positively on the executive functions (including set-shifting, planning and categorization), frequently altered in PSP.[43]

The analysis of our data let us to conclude that Lokomat® does not add further benefits within a protocol like MIRT. Moreover, the Lokomat® has a limitation in the maximum belt speed (equal to 3.2 km/h), while the treadmill-plus has not. It is therefore arguable that better results could be obtained with the treadmill plus by further increasing the speed of the belt.

Our data suggest that aerobic, motor-cognitive and goal-based rehabilitation treatments based on a multidisciplinary and intensive approach are useful for PSP patients, even without the support of expensive robotic technologies such as Lokomat®. Nevertheless, it is conceivable that Lokomat® training could be indicated for patients with those severe balance and/or walking dysfunctions that limit the use of treadmill.

## Study limitations

There are several limitations of this study that need to be acknowledged. First, the lack of a control group of not treated patients does not allow to draw definite conclusions on the effectiveness of both rehabilitation protocols. Nonetheless, a comparison of the observed improvements with the 9.7 points per year rate of progression of total PSPRS in untreated PSP patients^28^ indicates that our results are promising. Second, patients with cognitive impairments were included in the study and this might have affected the results. Another potential limitation of our study is the choice of a sample size of convenience, due to time constraints in carrying out the enrollment of patients. The relatively small number of patients might have determined a lack of statistical power in assessing differences in the efficacy of the two rehabilitation protocols. We should also consider that, being both treatments multidisciplinary and intensive, no large differences in their effects could be observed. The lack of follow-up data is another limitation of this study, since it is unknown how long the gains obtained during the rehabilitation period were sustained after discharge in the two groups of patients.

We reported a reduction in the number of falls during the last week of hospitalization. Even if this represents a very interesting finding, it may be at least in part related to the inpatient setting, where the health care personnel observe and monitor the patients continuously. Finally, the found improvements are defined only by the use of clinical and functional scales, without collecting any instrumental-quantitative data. Further studies are needed to clarify the issues not analyzed in this study.

## Conclusions

We showed that the use of Lokomat®, compared to the treadmill-plus training, does not add any further benefit in the context of an aerobic, multidisciplinary, intensive, motor-cognitive and goal-based rehabilitation treatment in PSP patients. Our data confirm the results from previous studies about the beneficial effect of rehabilitation in PSP and emphasize the need to design more specific rehabilitation programs for this group of patients.

## Supporting information

S1 Table Demographic and clinical characteristics of patients assigned to MIRT group and MIRT-Lokomat group. (DOCX)

S2 Table Baseline and end of treatment values of outcome variables for both groups of patients. (DOCX)

S3 Table Difference (discharge-admission) of the outcome variables for MIRT+Lokomat group and MIRT group and effect size. (DOCX)

S1 File Consort 2012 checklist. (DOC)

S2 File Trial study protocol italian (original). (DOC)

S3 File Trial study protocol italian (orginal). (PDF)

S4 File Trial study protocol english (translation). (DOC)

S5 File Trial study protocol English (translation). (PDF)
