# Supplementary material for: Rehabilitation in progressive supranuclear palsy: Effectiveness of two multidisciplinary treatments
Source: PLoS One. 2017 Feb 3;12(2):e0170927. doi: 10.1371/journal.pone.0170927 (PMC5291505; doi:10.1371/journal.pone.0170927)
Supplement: S1 Table — (DOCX) [file pone.0170927.s001.docx]

**Table 1. Demographic and clinical characteristics of patients assigned to MIRT group and MIRT-Lokomat group.** Reported p-values are computed by the Chi-square test for the variable Sex, by unpaired t-test for age, LED, weight, height, disease duration and by the Mann–Whitney U test for all the other variables. Data are reported as median (lower quartile, upper quartile) or mean±SD for non-normally and normally data respectively and as number (frequency percentage) for discrete variables.

| **Variable** | **MIRT-Lokomat group** | **MIRT group** | **p-value** |
| --- | --- | --- | --- |
| **Age (years) Range** | 69.9±5.2 60-77 | 72.5±6.1 64-83 | 0.28 |
| **LED (mg/die)** | 274.2 ± 217.9 | 375.8 ± 254.5 | 0.31 |
| **Sex (% Male)** | 41 | 58 | 0.41 |
| **Weight (Kg)** | 71.4±5.3 | 71.9±6.4 | 0.84 |
| **Height (cm)** | 169±7.1 | 168±5.9 | 0.84 |
| **Disease duration (yrs)** | 4.1 ± 1.4 | 4.0 ± 1.2 | 0.88 |
| **MMSE** | 25.6±1.94 | 25.1±3.87 | 0.69 |
| **FAB** | 11.7±3.7 | 10.5±2.48 | 0.46 |
| **PSPRS-Total** | 35.0 (29.0,44.5) | 34.0 (25.0,42.0) | 0.47 |
| **PSPRS-limb** | 5.50 (3.50,6.00) | 5.00 (3.50,6.50) | 0.98 |
| **PSPRS-gait** | 12.0 (9.0,13.0) | 10.5 (8.5,13.5) | 0.91 |

Abbreviations: MIRT (Multidisciplinary Intensive Rehabilitation Treatment); LED (levodopa equivalent dose), FAB (Frontal Assessment Battery); MMSE (Mini Mental State Examination); PSPRS (Progressive Supranuclear Palsy Rating Scale).
