# Supplementary material for: Rehabilitation in progressive supranuclear palsy: Effectiveness of two multidisciplinary treatments
Source: PLoS One. 2017 Feb 3;12(2):e0170927. doi: 10.1371/journal.pone.0170927 (PMC5291505; doi:10.1371/journal.pone.0170927)
Supplement: S2 Table — (DOCX) [file pone.0170927.s002.docx]

**Table 2. Baseline and end of treatment values of outcome variables for both groups of patients.** Reported p-values are computed by the Wilcoxon signed rank test. Data are reported as median (lower quartile, upper quartile).

| **Variable** | **MIRT-Lokomat group T0** | **MIRT-Lokomat groupt T1** | **p-value** | **MIRT group T0** | **MIRT group T1** | **p-value** |
| --- | --- | --- | --- | --- | --- | --- |
| **PSPRS-Total** | 35.0 (29.0,44.5) | 31.5 (25.5,38.0) | 0.0005 | 34.0 (25.0,42.0) | 27.5 (19.0,32.0) | 0.0005 |
| **PSPRS-limb** | 5.50 (3.50,6.00) | 4.00 (3.00,5.00) | 0.076 | 5.00 (3.50,6.50) | 4.00 (2.00,4.00) | 0.0020 |
| **PSPRS-gait** | 12.0 (9.0,13.0) | 8.5 (7.0,10.0) | 0.0005 | 10.5 (8.5,13.5) | 7.5 (5.0,9.5) | 0.0005 |
| **BBS** | 30.0 (26.5,38.5) | 47.0 (35.5,51.5) | 0.0005 | 35.5 (25.0,43.5) | 49.0 (45.5,51.0) | 0.0005 |
| **6MWT** | 223 (189,282) | 270 (232,325) | 0.032 | 262 (176,322) | 276 (236,349) | 0.018 |
| **Number of Falls** | 8.00 (5.00,10.50) | 2.00 (1.00,2.00) | 0.0015 | 7.50 (5.50,10.50) | 2.00 (1.00,2.50) | 0.0029 |

Abbreviations: MIRT (Multidisciplinary Intensive Rehabilitation Treatment); PSPRS (Progressive Supranuclear Palsy Rating Scale); BBS (Berg Balance Scale); 6MWT (Six Minutes Walking test).
