# Supplementary material for: Rehabilitation in progressive supranuclear palsy: Effectiveness of two multidisciplinary treatments
Source: PLoS One. 2017 Feb 3;12(2):e0170927. doi: 10.1371/journal.pone.0170927 (PMC5291505; doi:10.1371/journal.pone.0170927)
Supplement: S3 Table — (DOCX) [file pone.0170927.s003.docx]

**Table 3. Difference (discharge-admission) of the outcome variables for MIRT+Lokomat group and MIRT group and effect size.** Reported p-values are computed by the Mann–Whitney U test.

| **Variable** | **delta – MIRT-Lokomat group** | **Effect size** | **delta – MIRT group** | **Effect size** | **p-value** |
| --- | --- | --- | --- | --- | --- |
| **PSPRS-total** | -5.00 (-5.50,-3.50) | -0.6 | -8.00 (-9.50,-5.00) | -0.6 | 0.047 |
| **PSPRS-limb** | -1.00 (-1.00,-0.50) | -0.4 | -1.50 (-2.00,-1.00) | -0.7 | 0.067 |
| **PSPRS-gait** | -3.00 (-4.00,-2.00) | -1.5 | -4.00 (-4.50,-3.00) | -1.3 | 0.17 |
| **BBS** | 9.50 (6.50,19.00) | 1.2 | 10.50 (8.00,20.50) | 1.4 | 0.40 |
| **6MWT** | 32.5 (19.5,99.5) | 0.5 | 55.5 (-3.0,67.5) | 0.4 | 0.62 |
| **Number of Falls** | -6.50 (-8.00,-3.50) | -2 | -5.50 (-9.00,-3.50) | -1.3 | 0.98 |

Abbreviations: MIRT (Multidisciplinary Intensive Rehabilitation Treatment); PSPRS (Progressive Supranuclear Palsy Rating Scale); BBS (Berg Balance Scale); 6MWT (Six Minutes Walking test).
