# Supplementary material for: Rehabilitation in progressive supranuclear palsy: Effectiveness of two multidisciplinary treatments
Source: PLoS One. 2017 Feb 3;12(2):e0170927. doi: 10.1371/journal.pone.0170927 (PMC5291505; doi:10.1371/journal.pone.0170927)
Supplement: S2 File — (DOC) [file pone.0170927.s005.doc]

# DIPARTIMENTO RIABILITAZIONE MALATTIA DI PARKINSON E DISTURBI DEL MOVIMENTO

# RESPONSABILE: Dr Frazzitta Giuseppe

*Gravedona ed Unitì lì, 21.11.2013*

**Al comitato Tecnico-Scientifico locale dell’Ospedale “Moriggia-Pelascini”, Via Pelascini 3, Gravedona ed Uniti – Como.**

**Studio proposto:** efficacia di un trattamento riabilitativo intensivo, aerobico-multidisciplinare e contributo dell’utilizzo del Lokomat® nella riabilitazione motoria dei pazienti affetti da paralisi sopranucleare progressiva.

**Investigatore Principale:** Dr. Giuseppe Frazzitta – Dipartimento di Neuroriabilitazione Malattia di Parkinson, Disturbi del Movimento e Gravi Cerebrolesioni, Osepdale Generale di Zona Moriggia-Pelascini, Gravedona ed Uniti - CO.

**Introduzione e Razionale**

La **paralisi sopranucleare progressiva (PSP)** o Sindrome di *Steele-Richardson-Olszewski* è una malattia neurodegenerativa descritta per la prima volta nel 1964. La PSP condivide alcuni aspetti neuropatologici e fisiopatologici in comune con la malattia di Parkinson (MP) pur rappresentando per se stessa una condizione indipendente. La neurodegenerazione nella PSP comporta [atrofia](http://it.wikipedia.org/wiki/Atrofia) a livello del [mesencefalo](http://it.wikipedia.org/wiki/Mesencefalo) e di altre strutture cerebrali tra cui il [nucleo subtalamico](http://it.wikipedia.org/wiki/Nucleo_subtalamico), il [globus pallidus](http://it.wikipedia.org/wiki/Globus_pallidus), i nuclei del [ponte](http://it.wikipedia.org/wiki/Ponte_di_Varolio) e la [sostanza nera](http://it.wikipedia.org/wiki/Sostanza_nera). I sintomi principali della PSP sono:

- Marcata instabilità posturale con frequenti cadute soprattutto all’indietro;
- Disturbi dell’oculomotricità con paralisi dello sguardo prevalentemente verso l’alto;
- Disturbi neuropsicologici;

Trascorso un intervallo medio di circa 3,9 anni dall’esordio dei disturbi neurologici, si delinea un quadro clinico tale da permette la diagnosi dell'affezione. Non esistono terapie mediche efficaci per la cura della PSP. Questa condizione determina rapidamente grave disabilità e nella maggior parte dei casi la morte avviene dopo 6-7 anni circa dall’esordio della sintomatologia.

Percorsi riabilitativi aerobici e *goal-based* basati sulla multidisciplinarietà, l’intensità e la ripetizione si sono già dimostrati efficaci nel controllo e nel trattamento dei disturbi motori nei pazienti affetti da MP, ma l’efficacia di questi approcci sui pazienti affetti da PSP non è mai stata valutata. Questo in parte deriva dalla difficoltà che s’incontra nel lavorare con pazienti affetti da questa condizione e dalle criticità che derivano dal trasferire percorsi riabilitativi costruiti per la MP a pazienti con PSP.

La deambulazione nei pazienti affetti da PSP è marcatamente alterata e conduce a instabilità: durante la marcia la base di appoggio è allargata, c’è una forte tendenza alla retropulsione e instabilità posturo-cinetica con perdita dei normali riflessi posturali di raddrizzamento. Partendo da queste basi ipotizziamo che un *device* robotizzato come il Lokomat® possa intervenire positivamente sui meccanismi di controllo deambulatori e posturali così marcatamente alterati nella PSP.

Il Lokomat® è un supporto robotico per la deambulazione, dotato di un esoscheletro e collegato ad un computer. Permette simultaneamente di scaricare (in parte o in toto) il peso corporeo del paziente e assiste in modo motorizzato il suo cammino. L’operatore può impostare i parametri del cammino controllati dall’esoscheletro robotizzato (velocità, lunghezza del passo, escursione articolare del ginocchio e delle anche) sulla base delle necessità del singolo paziente. Il dispositivo fornisce anche dei *feedbacks* visivi su uno schermo che possono essere utilizzati dal paziente per interagire in modo “attivo” con i movimenti passivi indotti dall’esoscheletro.

Visti questi presupposti e l’esistenza di dati di letteratura che supportano l’utilizzo di altre apparecchiature robotiche per il training dell’equilibrio e del cammino, noi riteniamo che il Lokomat® possa costituire un valido supporto alla terapia riabilitativa nei pazienti affetti da PSP.

Resta tuttavia in primo luogo da chiarire l’efficacia di approcci riabilitativi già validati su popolazioni di pazienti parkinsoniani nel contesto della riabilitazione della PSP. Il trattamento conosciuto come MIRT (*Multidisciplinary Intensive Rehabilitation Treatment*), già estensivamente descritto, si è dimostrato efficace sui parametri motori e funzionali nei pazienti affetti da MP. Questo trattamento **mira al recupero funzionale e al ri-apprendimento motorio,** ha una durata di 4 settimane, ciascuna strutturata in 5 giorni di lavoro (dal lunedì al venerdì) così organizzati:

- *Lunedì-venerdì 🡪* ***1° sessione****, fisiokinesiterapia front-to-front (1 ora/die);* ***2° sessione****, esercizi aerobici e con utilizzo di devices meccanici (Treadmill-plus con cues visivi e uditivi, Cicloergometro, Crossover, Pedana stabilometrica) (1 ora/die);* ***3° sessione****, terapia occupazionale (1 ora/die);* ***4°sessione,*** *logopedia (1 ora/die);*

**Obiettivi dello studio**

Gli obiettivi del nostro studio sono i seguenti:

- Valutare l’efficacia del MIRT sui pazienti affetti da PSP;
- Valutare se l’utilizzo del Lokomat® associato al MIRT (MIRT+Lokomat) garantisce ulteriori benefici rispetto al solo trattamento MIRT;

**Materiali e Metodi**

Nel periodo compreso tra gennaio 2014 e dicembre 2015 i pazienti con diagnosi di PSP che afferiranno presso il Dipartimento di Neuroriabilitazione Malattia di Parkinson, Disturbi del Movimento e Gravi Cerebrolesioni dell’Ospedale Moriggia-Pelascini di Gravedona ed Uniti (CO), verranno valutati per partecipare allo studio.

Saranno considerati eleggibili e quindi arruolabili per lo studio (previa firma di consenso informato) quei pazienti che soddisferanno i seguenti **criteri di inclusione**:

- Diagnosi di PSP in accordo con i criteri internazionali NINDS-SPSP (*Litvan et al. 1996*);
- Età minima 55 anni e massima 85 anni;
- Capacità di camminare senza assistenza per almeno 6 metri;
- Dosaggio stabile della terapia dopaminergica nel mese precedente l’arruolamento nello studio;

**Criteri di esclusione**:

- Sovrimposizione di altre condizioni neurologiche e/o ortopediche;
- Presenza di osteoartrite, osteoporosi, lesioni cutanee e altre ferite da pressione;
- Peso corpo superiore a 135 kg (peso massimo consentito per l’utilizzo del Lokomat®);
- Presenza di malattie cardiovascolari e respiratorie;

**I pazienti arruolati saranno randomizzati tramite software dedicato e quindi assegnati a due gruppi:**

**Misure di Outcome**

Entrami i gruppi di pazienti saranno valutati all’inizio (T0) e al termine del trattamento riabilitativo (T1) da neurologi e fisioterapisti esperti in disordini del movimento utilizzando le seguenti misure di outcome:

- Misura di outcome primario 🡪 PSP rating scale (PSPRS);
- Misure di outcome secondario 🡪 a) Berg Balance Scale (BBS); b) Numero di Cadute (NoF); c) Six-Minutes Walking Test (6MWT);

**Analisi Statistica**

La normalità della distribuzione delle variabili sarà valutata attraverso lo Shapiro-Wilk test. In caso di distribuzione non normale dei dati i confronti tra gruppi ed inter-gruppo saranno effettuati utilizzando rispettivamente il Mann-Whitney U Test e il Wilcoxon *signed-rank* test. Il t-test accoppiato e non accoppiato sarà utilizzato per le variabili normalmente distribuite. Per determinare se l’utilizzo del Lokomat® nel gruppo MIRT+Lokomat determina miglioramenti aggiuntivi rispetto al MIRT per ogni misura di outcome sarà calcolata la differenza (T1-T0) e saranno effettuati test non parametrici per valutare l’effetto del trattamento.

Tutti i test statistici sono a due code e la significatività è fissata per p < 0.05.

**Referenze**

Schrag A, Ben-Shlomo Y, Quinn NP. Prevalence of progressive supranuclear palsy and multiple system atrophy: a cross-sectional study. Lancet. 1999;354: 1771-1775.

Steele JC, Richardson JC, Olszewski J. Progressive supranuclear palsy. a heterogeneous degeneration involving the brain stem, basal ganglia and cerebellum with vertical gaze and pseudobulbar palsy, nuchal dystonia and dementia. Arch Neurol. 1964;10: 333-359.

Zampieri C, Di Fabio RP. Progressive supranuclear palsy: disease profile and rehabilitation strategies. Phys Ther. 2006;866: 870-880.

Litvan I, Mangone CA, McKee et al. Natural history of progressive supranuclear palsy (Steele-Richardson-Olszewski syndrome) and clinical predictors of survival: a clinicopathological study. J Neurol Neurosurg Pshychiatry. 1996;60: 615-620.

Frazzitta G, Maestri R, Uccellini D, Bertotti G, Abelli P. Rehabilitation treatment of gait in patients with Parkinson's disease with freezing: a comparison between two physical therapy protocols using visual and auditory cues with or without treadmill training. Mov Disord. 2009;24: 1139-1143.

Frazzitta G, Bertotti G, Uccellini D. et al. Parkinson’s disease rehabilitation: a pilot study with 1 year follow up. Mov Disord. 2010;25: 1762-1763.

Frazzitta G, Balbi P, Maestri R, Bertotti G, Boveri N, Pezzoli G. The beneficial role of intensive exercise on Parkinson disease progression. Am J Phys Med Rehabil*.* 2013;92: 523-532.

Petzinger GM, Fisher BE, Van Leeuwen JE. Enhancing neuroplasticity in the basal ganglia: the role of exercise in Parkinson's disease. Mov Disord. 2010;25: S141-145.

Golbe LI, Ohman-Strickland PA. A clinical rating scale for progressive supranuclear palsy. Brain. 2007;130: 1552-15565.

Guyatt GH, Sullivan MJ, Thompson PJ et al. The 6-minute walk: a new measure of exercise capacity in patients with chronic heart failure. Can Med Assoc J. 1985;132: 919-923.

Lach HW, Reed AT, Arfken CL et al. Falls in the elderly: reliability of a classification system. J Am Geriatr Soc. 1991;39: 197-202.

Cakit BD, Saracoglu M, Genc H, Erdem HR, Inan L. The effects of incremental speed-dependent treadmill training on postural instability and fear of falling in Parkinson's disease. Clin Rehabil*.* 2007;21: 698-705.

Tool T, Maitland CG, Warren E, Hubmann MF, Panton L. The effects of loading and unloading treadmill walking on balance, gait, fall risk, and daily function in Parkinsonism. NeuroRehabilitation. 2005;20: 307-322.

Hohler AD, Tsao JM, Kats DI et al. Effectiveness of an inpatient movement disorders program for patients with atypical parkinsonism. Parkinsons Dis. 2012;2012: 871974.
