# Supplementary material for: Rehabilitation in progressive supranuclear palsy: Effectiveness of two multidisciplinary treatments
Source: PLoS One. 2017 Feb 3;12(2):e0170927. doi: 10.1371/journal.pone.0170927 (PMC5291505; doi:10.1371/journal.pone.0170927)
Supplement: S4 File — (DOC) [file pone.0170927.s007.doc]

# DIPARTIMENTO RIABILITAZIONE MALATTIA DI PARKINSON E DISTURBI DEL MOVIMENTO

# RESPONSABILE: Dr Frazzitta Giuseppe

*Gravedona ed Uniti, Como - November 21, 2013*

**To the local Scientific Committee and Institutional Review Board of the ‘Moriggia-Pelascini’ Hospital, Gravedona ed Uniti, Como.**

**Proposed Study:** effectiveness of an intensive, aerobic and multidisciplinary rehabilitation treatment and evaluation of the contribution of Lokomat® for the rehabilitation of patients suffering from Progressive Supranuclear Palsy.

**Principal Investigator:** Giuseppe Frazzitta, MD – Dipartimento di Neuroriabilitazione Malattia di Parkinson, Disturbi del Movimento e Gravi Cerebrolesioni, Osepdale Generale di Zona Moriggia-Pelascini, Gravedona ed Uniti - CO.

**Introduction and rationale of the study**

**Progressive Supranuclear Palsy** (PSP) (also known as *Steele-Richardson-Olszewski* Syndrome) is a neurodegenerative disorder first described in 1964. PSP shares different neuropathological and neurophysiological aspects in common with Parkinson’s Disease (PD) and some data provide insight into a common deficit in both pathologies. A biochemical alteration in the tau protein, resulting in neurodegeneration and gliosis in the basal ganglia, brainstem, prefrontal cortex and cerebellum, is the neuropathological hallmark of PSP. The principal clinical features of PSP are:

- Early postural instability with recurrent falls (principally backwards);
- Visual dysfunctions (vertical supranuclear gaze palsy);
- Neuropsychological deficits;

After about 3.9 years from onset of the disturbances, the clinical picture becomes particularly distinctive. To date, there are no effective medical or surgical treatments for this disease. Patients rapidly become disabled and death generally occurs after about 6-7 years from the onset of the disorder.

Literature data support the efficacy of *goal-based*, aerobic, intensive and multidisciplinary rehabilitation treatments for PD patients. Nevertheless, data about the effectiveness of these physical approaches in PSP patients are not consistent and basic questions remain to clarify. As a matter of fact, the severe gait and balance disturbances, together with the high risk of falls, limit the design of specific rehabilitation protocols for PSP patients.

Gait is heavily impaired in PSP and causes imbalance: while walking the base of support is augmented, there is an important trunk retropulsion, posturo-kinetic instability and loss of the physiological postural reactive reflexes.

Given these premises, we hypothisize that the use of a robotic device such as Lokomat® could positively intervene on the typical gait and balance disturbances of PSP. Lokomat® is a driven-gait orthosis (gait robot) that allows gait by simulating physiological stride patterns and inducing a muscle coordination reflecting human walking. This robotic device is connected to a computer: the operator can set the gait parameters driven by the robotic orthosis (speed, stride length, range of motion of the knee and hips) on the basis of the clinical feature of each patient. Patients, in order to “actively” interact with the passive robot-driven movements, could use visual feedbacks provided by the computer. Therefore, it is arguable that Lokomat® could be a valid support for the rehabilitation of PSP patients. Anyhow it remains to be clarified the effectiveness of rehabilitative approaches designed for PD patients on subjects suffering from PSP.

The rehabilitative treatment known as MIRT (*Multidisciplinary Intensive Rehabilitation Treatment*) has been extensively described and it its effectiveness on motor and functional parameters has been demonstrated in PD patients. MIRT aims at functional recovery and motor re-learning. It consists of a 4-week physical therapy in a hospital setting and entails four daily sessions for five days per week organized as follows:

- **From Monday to Friday** 🡪 ***First session (1 h/day)****: it consists of a one-to-one session with physical therapist (front-to-front);* ***Second session (1 h/day)****: it includes aerobic and repetitive activities to improve gait and balance using different devices (treadmill-plus with visual cues and auditory feedbacks, cycloergometer, crossover and posturographic platform with visual feedbacks);* ***Third sessions (1 h/day)****: occupational therapy;* ***Fourth session (1 h/day)****: speech therapy*.

**Aims of the study**

The aims of our study are:

- To evaluate whether an aerobic, intensive, goal-based and multidisciplinary rehabilitation treatment such as MIRT, previously conceived for PD, is effective for PSP patients;
- To evaluate, within MIRT, whether a robotic device, such as Lokomat®, is able to provide further benefits;

**Materiali e Metodi**

From January 2014 to December 2015, patients with a diagnosis of PSP, which will be hospitalized at the Department of Parkinson’s Disease, Movement Disorders and Brain Injury Rehabilitation of the “Moriggia-Pelascini” Hospital (Gravedona ed Uniti, Italy), will be evaluated to be enrolled in the study.

**Inclusion criteria:**

- Diagnosis of PSP in accordance to the NINDS-SPSP International Criteria (*Litvan et al., 1996*);
- Ability to walk unassisted for at least 6 meters;
- Age 55-85;
- Stable dopaminergic drugs dosage in the month preceding the admission to the study;

**Exclusion criteria:**

- Any others significant neurological or orthopedic disorders;
- Osteoarthritis, osteoporosis, cutaneous lesions and/or other pressure wounds;
- Body weight exceeding 135 kg (the weight limit for the use of Lokomat®);
- Respiratory and cardiovascular diseases;

**Written informed consent will be requested to participants before the beginning of the study.**

**Patients will be randomly assigned to two groups (each group was composed of 12 patients) using a computer-generated list:**

**Outcome measures**

Both groups will be evaluated at admission (T0) and discharge (T1) by neurologists and physiotherapists with expertise in movement disorders field, using the following outcome measures:

- Primary outcome measure 🡪 PSP rating Scale (PSPRS);
- Secondary outcome measures 🡪 a) Berg Balance Scale (BBS); b) Number of Falls (NoF); c) Six-Minutes Walking Test (6MWT);

**Statistical Analysis**

The normality of the distribution of all variables will be assessed by the Shapiro–Wilk test. For non-normally distributed variables, between- and within-group comparisons will be performed by the Mann-Whitney U test and Wilcoxon signed-rank test respectively. The unpaired and paired t-tests will be used for normally distributed variables. To assess whether, within our intensive and aerobic treatment, the use of Lokomat® in the MIRT-Lokomat protocol could lead to a better improvement as compared to MIRT, for each outcome variable we will compute the difference (discharge-admission) and then run the non parametric test on the treatment factor. All statistical tests are two-tailed and statistical significance are set at p < 0.05.

**References**

Schrag A, Ben-Shlomo Y, Quinn NP. Prevalence of progressive supranuclear palsy and multiple system atrophy: a cross-sectional study. Lancet. 1999;354: 1771-1775.

Steele JC, Richardson JC, Olszewski J. Progressive supranuclear palsy. a heterogeneous degeneration involving the brain stem, basal ganglia and cerebellum with vertical gaze and pseudobulbar palsy, nuchal dystonia and dementia. Arch Neurol. 1964;10: 333-359.

Zampieri C, Di Fabio RP. Progressive supranuclear palsy: disease profile and rehabilitation strategies. Phys Ther. 2006;866: 870-880.

Litvan I, Mangone CA, McKee et al. Natural history of progressive supranuclear palsy (Steele-Richardson-Olszewski syndrome) and clinical predictors of survival: a clinicopathological study. J Neurol Neurosurg Pshychiatry. 1996;60: 615-620.

Frazzitta G, Maestri R, Uccellini D, Bertotti G, Abelli P. Rehabilitation treatment of gait in patients with Parkinson's disease with freezing: a comparison between two physical therapy protocols using visual and auditory cues with or without treadmill training. Mov Disord. 2009;24: 1139-1143.

Frazzitta G, Bertotti G, Uccellini D. et al. Parkinson’s disease rehabilitation: a pilot study with 1 year follow up. Mov Disord. 2010;25: 1762-1763.

Frazzitta G, Balbi P, Maestri R, Bertotti G, Boveri N, Pezzoli G. The beneficial role of intensive exercise on Parkinson disease progression. Am J Phys Med Rehabil*.* 2013;92: 523-532.

Petzinger GM, Fisher BE, Van Leeuwen JE. Enhancing neuroplasticity in the basal ganglia: the role of exercise in Parkinson's disease. Mov Disord. 2010;25: S141-145.

Golbe LI, Ohman-Strickland PA. A clinical rating scale for progressive supranuclear palsy. Brain. 2007;130: 1552-15565.

Guyatt GH, Sullivan MJ, Thompson PJ et al. The 6-minute walk: a new measure of exercise capacity in patients with chronic heart failure. Can Med Assoc J. 1985;132: 919-923.

Lach HW, Reed AT, Arfken CL et al. Falls in the elderly: reliability of a classification system. J Am Geriatr Soc. 1991;39: 197-202.

Cakit BD, Saracoglu M, Genc H, Erdem HR, Inan L. The effects of incremental speed-dependent treadmill training on postural instability and fear of falling in Parkinson's disease. Clin Rehabil*.* 2007;21: 698-705.

Tool T, Maitland CG, Warren E, Hubmann MF, Panton L. The effects of loading and unloading treadmill walking on balance, gait, fall risk, and daily function in Parkinsonism. NeuroRehabilitation. 2005;20: 307-322.

Hohler AD, Tsao JM, Kats DI et al. Effectiveness of an inpatient movement disorders program for patients with atypical parkinsonism. Parkinsons Dis. 2012;2012: 871974.
